# Age-dependent effects of body mass index across the adult life span on the risk of dementia: a cohort study with a genetic approach

**DOI:** 10.1186/s12916-020-01600-2

**Published:** 2020-06-09

**Authors:** Ida K. Karlsson, Kelli Lehto, Margaret Gatz, Chandra A. Reynolds, Anna K. Dahl Aslan

**Affiliations:** 1 Institute of Gerontology and Aging Research Network – Jönköping (ARN-J), School of Health and Welfare, Jönköping University, Box 1026, SE-551 11 Jönköping, Sweden; 2 Department of Medical Epidemiology and Biostatistics, Karolinska Institutet, Stockholm, Sweden; 3 National Institute for Health Development, Tallinn, Estonia; 4 Department of Psychology, University of Southern California, Los Angeles, CA USA; 5 Department of Psychology, University of California, Riverside, USA

**Keywords:** Body mass index, Dementia, Obesity paradox, Polygenic score, Twin design, Life span, Longitudinal

## Abstract

**Background:**

While a high body mass index (BMI) in midlife is associated with higher risk of dementia, high BMI in late-life may be associated with lower risk. This study combined genetic designs with longitudinal data to achieve a better understanding of this paradox.

**Methods:**

We used longitudinal data from 22,156 individuals in the Swedish Twin Registry (STR) and 25,698 from the Health and Retirement Study (HRS). The STR sample had information about BMI from early adulthood through late-life, and the HRS sample from age 50 through late-life. Survival analysis was applied to investigate age-specific associations between BMI and dementia risk. To examine if the associations are influenced by genetic susceptibility to higher BMI, an interaction between BMI and a polygenic score for BMI (PGS_BMI_) was included in the models and results stratified into those with genetic predisposition to low, medium, and higher BMI. In the STR, co-twin control models were applied to adjust for familial factors beyond those captured by the PGS_BMI_.

**Results:**

At age 35–49, 5 units higher BMI was associated with 15% (95% CI 7–24%) higher risk of dementia in the STR. There was a significant interaction (*p* = 0.04) between BMI and the PGS_BMI_, and the association present only among those with genetic predisposition to low BMI (HR 1.38, 95% CI 1.08–1.78). Co-twin control analyses indicated genetic influences. After age 80, 5 units higher BMI was associated with 10–11% lower risk of dementia in both samples. There was a significant interaction between late-life BMI and the PGS_BMI_ in the STR (*p* = 0.01), but not the HRS, with the inverse association present only among those with a high PGS_BMI_ (HR 0.70, 95% CI 0.52–0.94)_._ No genetic influences were evident from co-twin control models of late-life BMI.

**Conclusions:**

Not only does the association between BMI and dementia differ depending on age at BMI measurement, but also the effect of genetic influences. In STR, the associations were only present among those with a BMI in opposite direction of their genetic predisposition, indicating that the association between BMI and dementia across the life course might be driven by environmental factors and hence likely modifiable.

## Background

Over the last decades, we have seen a substantial increase in obesity in both developed and developing countries, with the prevalence reaching pandemic proportions [1]. As a high body mass index (BMI) is associated with many age-related diseases, this increase in obesity prevalence, together with the demographic shift in the proportion of older individuals, calls for substantial efforts to better understand how BMI affects long-term health outcomes.

BMI is associated with dementia, but research indicates that the relationship differs depending on the age when BMI is measured [2]. While a high BMI in midlife has been associated with increased risk of dementia in most studies, a high BMI in late-life may in fact be associated with a decreased risk of dementia—this is known as the “obesity paradox” [3]. One hypothesis regarding the nature of this paradox is that unintentional weight loss in late-life may be an early sign in the prodromal stage of dementia [2].

BMI and dementia are both complex phenotypes, influenced by both genetic and environmental factors. According to twin studies, as much as 60–80% of the variance in late-onset Alzheimer’s disease (AD), the most common form of dementia, is explained by genetic factors [4]. The twin-based heritability of BMI has been estimated to 45–85% [5], and 941 genetic variants have been identified in the most recent genome-wide association study (GWAS) [6]. Several studies, using different methods, have shown that the genetic influences of BMI differ across age groups. A meta-analysis of twin studies showed that the heritability of BMI gradually decreased from its peak around age 20 to age 55, after which it gradually increased again [5]. A GWAS identified several genetic variants with age-specific effects on BMI, most of which are more important for BMI prior to age 55 than at later ages [7]. In line with these latter findings, Song et al. showed that while a genetic score comprised of 97 genetic variants for BMI was associated with BMI across all age categories, the effect declined in late-life [8]. Thus, not only the negative health effects associated with higher BMI differ by age at BMI measurement, but also the genetic influences and thus the underlying biological mechanisms.

Genetic study designs can be valuable tools in understanding biological mechanisms underlying diseases and phenotypic associations. However, in the case of BMI and dementia, this is complicated by the age-specific effects of BMI, and to date, very little work has been done on the subject. Thus, we aimed to study how genetic factors influence the age-dependent effects of BMI on the risk of dementia, from early adulthood through late-life. Hence, we utilized genetic designs combined with large longitudinal data, stratifying the results on age at BMI measurements, and further by genetic predisposition to higher BMI. We could thus study how genetic influences change across age and indicate whether the age-specific effects of BMI on the risk of dementia are driven directly by BMI level, or better explained by other genetic or environmental factors.

## Methods

### Study population

The study population is based on longitudinal data from two sources: studies of aging within the Swedish Twin Registry (STR) [9] and the Health and Retirement Study (HRS) [10]. A flowchart of the sample and exclusions made can be found in Additional file 1: Figure S1.

#### The Swedish Twin Registry

The STR is a population-based register of twins born in 1886–2008 [9]. The current study population stems from five sub-studies of aging within the STR. The Swedish Adoption/Twin Study of Aging (SATSA) [11] is a longitudinal study of 859 individuals from same-sex twin pairs, consisting of up to 10 in-person testing waves conducted across 30 years (1986–2014). Aging in Women and Men (GENDER) [12] was initiated in 1995 and is a study of 248 opposite-sex twin pairs, with up to three in-person testing occasions on a 4-year rolling schedule. Origins of Variance in the Oldest Old: Octogenarian Twins (OCTO-Twin) [13] is a longitudinal study focused on twins over the age of 80 and includes 351 same-sex twin pairs. The study was initiated in 1991 and consists of up to five in-person testing waves conducted every 2 years. The Study of Dementia in Swedish Twins (HARMONY) [14] is a cross-sectional census of all twins aged 65 or older conducted 1998–2003, entailing a telephone screening for cognitive dysfunction. All twins who screened positive for cognitive dysfunction, as well as their co-twins and a control sample, were referred to a complete clinical examination. In total, 13,939 individuals participated in the screening phase and 1557 in the clinical examination. TwinGene [9] was conducted 2004–2008 and is a cross-sectional study of 12,630 twins born before 1958, who answered a questionnaire and underwent a health checkup.

In total, the sample consisted of 24,823 individuals (some participated in more than one of the sub-studies).

#### The Health and Retirement Study

The HRS [10] is an open-access data source funded by the National Institute of Health. It includes data on more than 37,000 individuals over the age of 50 and residing in the USA, with follow-up on a biannual basis. The study was initiated in 1992 and now includes up to 13 follow-up occasions. The HRS has a steady-state design, with younger samples recruited every 6 years. African American and Hispanic households are oversampled to allow for a more diverse sample. The baseline interviews have mostly been conducted face-to-face. Up until 2004, the follow-up interviews were offered as face-to-face interviews only to participants over the age of 80, and otherwise conducted over the telephone. From the 2006 wave and onwards, half of the sample is assigned a face-to-face interview with physical and biological measures as well as a psychosocial questionnaire, while the other half completes the core interview over telephone. These then alternate between waves, so that participants go through the extended face-to-face interview every 4 years.

This study is based on the RAND HRS Longitudinal File 2014 (V3), an easy-to-use dataset based on the HRS core data, supplemented with data on dementia and PGS.

### BMI measurements

In the STR, height and weight were measured at each testing occasion. In addition, for same-sex twin pairs, self-reported height and weight were available from questionnaires conducted in the 1960s and 1970s, as well as retrospective height and weight at age 25 and 40. Twins born 1925 or earlier were sent questionnaires in 1961, 1963, 1967, and 1970. Twins born 1926–1958 were sent a questionnaire in 1973. BMI information was thus available from early adulthood though late-life for same-sex twin pairs. Additional file 1: Figure S2 shows an overview of STR data collections including BMI information. In the HRS, height and weight were measured at the baseline and extended face-to-face interview, and weight self-reported in the telephone interviews. Hence, BMI is available on a biannual basis in the HRS.

BMI was calculated as kilograms per square meter. For additional information about removing of outliers, please see Additional file 1: BMI data cleaning. To study age-specific effects, BMI was categorized according to age at measurement/reporting into age 20–34 (early adulthood), 35–49 (adulthood and early midlife), 50–64 (midlife), 65–79 (early late-life), and 80 and above (late-life). The STR sample contributed information to all age categories, and the HRS to the 50–64, 65–79, and 80 and above categories. Prior to analyses, BMI was mean centered within each age category and divided by 5, so that estimates correspond to the effect of 5 units higher BMI. The number of BMI measurements per age category, along with mean BMI, is presented in Additional file 1: Table S1.

### Dementia ascertainment

In the STR, dementia information was available both from clinical evaluations [14] part of the SATSA, OCTO-Twin, GENDER, and HARMONY studies (*n* = 15,016) and from nationwide registers. Registers used in this study were the National Patient Register (NPR), the Cause of Death Register (CDR), and the Prescribed Drug Register (PDR). The NPR and CDR include information about diseases classified according to the International Classification of Diseases (ICD) system. When diagnoses from the NPR and CDR are combined, the sensitivity for prevalent dementia is 63%, and the specificity is over 98% [15]. Across clinical diagnoses and registry linkage codes, Alzheimer’s disease accounted for 60% of dementia. The PDR includes information about dispensed medications according to Anatomical Therapeutic Chemical (ATC) codes, and dementia medication was used as proxy for diagnosis. All medications within the ATC categories N06D (anti-dementia drugs) were included (except Tacrine, Ipidacrine, and Ginko folium which are not prescribed in Sweden). More information on the registries and the clinical work-up is reported in Additional file 1: Dementia data in the Swedish Twin Registry. The ICD and ATC codes used are listed in Additional file 1: Table S2 and S3.

For the HRS data, we used the Langa-Weir classifications of dementia [16, 17]. Briefly, for HRS self-responders, the authors generated a 27-point scale based on the immediate and delayed 10-noun free recall test, the serial 7 subtraction test, and the backward count from 20 test. Cut points to categorize individuals into cognitively intact (score of 12 or higher), cognitive impairment, no dementia (CIND, score of 7–11), or dementia (score of 6 or lower) were validated against the subsample of HRS participating in the Aging, Demographics, and Memory Study (ADAMS). For HRS participants represented by a proxy, an 11-point scale was generated based on the proxy’s assessment of the respondent’s memory and limitations in daily living, and the interviewer’s assessment of whether the participant had problems completing the interview due to cognitive limitations. Participants given a score of 3–5 were classified as CIND, and those given a score of 0–2 classified with dementia. The validity of the definition has been investigated, resulting in a sensitivity of 62%, a specificity of 82%, and an accuracy of 79%, using unweighted validation data [18].

### Polygenic scores

Genotype data were available for 12,636 individuals in the STR sample and used to compute polygenic scores (PGSs) for BMI and AD which were used as a measure of genetic susceptibility to the trait. The PGSs were computed in Plink 1.9, according to the pipeline described in Additional file 1: PGS calculation in the Swedish Twin Registry. The PGS for BMI (PGS_BMI_) was based on GWAS summary statistics from the most recent GWAS for BMI from the GIANT consortium [6], which includes around 700,000 individuals of European ancestry. The STR studies were included in the BMI GWAS; thus to avoid inflation in the explanatory power of the PGS due to overlapping samples [19], new GWAS summary statistics were first generated by meta-analyzing the results, excluding those from the STR. The PGS for AD (PGS_AD_) was based on the most recent work from the IGAP consortium [20], using a pathology confirmed AD diagnosis in 21,982 cases and 41,944 controls. Nine PGSs were generated for each trait, based on *p* value thresholds ranging from *p* = 5 × 10^−8^ to *p* = 1. For BMI, the PGS with all independent variants with *p* < 0.5 best predicted BMI in the sample and was used in analyses. For AD, the PGS with *p* < 10^−5^ had the best predictive ability and was selected for analyses.

In the HRS sample, PGSs for various phenotypes, including BMI and AD, are available for 15,190 participants of European American (non-Hispanic whites, *n* = 12,090) and African American (*n* = 3100) descent [21]. The PGS_BMI_ was generated based on the second largest GWAS for BMI [22], which included 339,224 individuals. The PGS_AD_ was based on the previous IGAP publication [23], including 17,008 cases and 37,154 controls. It should be noted that the HRS sample was included in the BMI GWAS, and the PGS_BMI_ was generated based on GWAS summary statistics including the HRS sample. The pipeline for generating PGSs in the HRS includes all independent SNPs, regardless of significance level [21].

Prior to analyses, the PGSs were adjusted for ancestry and standardized within genotyping array (STR) or race (HRS), so that estimates represent the effect per standard deviation (SD) increase in PGS. In addition, a variable indicating genetic predisposition to low, medium, or high BMI was generated by categorizing individuals by tertiles of the PGS_BMI_.

### Statistical analyses

The effect of BMI on dementia was modeled using Cox proportional hazard model with age in years as the underlying timescale. The stratified Cox option was used to allow differences in the underlying hazard between sub-studies (STR) or ancestry (HRS). The different age categories of BMI measurement were analyzed separately, with multiple measures of BMI within the same category modeled as time-varying exposure. Individuals were followed from the first BMI measurement within each age category until death or end of follow-up, which was end of register follow-up for the STR sample (December 31, 2016) and last study participation for the HRS sample. In addition, HRS participants categorized as CIND were right-censored, i.e., followed from baseline until categorized as CIND (*n* = 8188).

For each age category, the following analyses were performed.

Firstly, a base model of BMI on the risk of dementia was modeled using (a) all samples and (b) samples with genotype data available. Next, to examine whether the associations are influenced by genetic predisposition to BMI, the PGS_BMI_ was introduced into the model. To investigate whether the effect remains after controlling for shared genetic etiology, both the PGS_BMI_ and the PGS_AD_ were then included in the model. In addition, to investigate whether the association differs depending on genetic predisposition to higher BMI, an interaction term between BMI and tertiles of the PGS_BMI_ was introduced to the base model, and the results could thus be stratified based on genetic predisposition to low, medium, and high BMI. Lastly, to study genetic factors beyond those captured by the PGS, co-twin control models were applied to the STR data using (a) all complete twin pairs, (b) dizygotic twin pairs, and (c) monozygotic twin pairs.

All models were adjusted for sex, education (basic vs more than basic education), and smoking (ever vs never smoking). Robust standard errors were used to account for clustering of individuals within twin pairs (STR) and households (HRS).

#### Sensitivity analyses

The analyses above were performed separately in men and women (STR and HRS), and individuals of European and African descent (HRS). To study the risk of dementia in the underweight, overweight, and obesity group compared to that in the normal weight group, BMI was categorized into underweight (< 18.5), normal weight (18.5–25), overweight (25–30), and obesity (> 30). Cox regression was then performed as above. To analyze the extent of survival bias in the findings, we performed competing risk analysis with dementia as the outcome and death as the competing risk. As in the main analyses, multiple measures of BMI within each age category were treated as a time-varying exposure, and models were adjusted as above.

All analyses were performed in STATA 15.1 [24].

## Results

### Study population

The STR analysis sample consisted of 22,156 individuals, out of whom 3732 developed dementia during the follow-up. The HRS analysis sample included 25,698 individuals, out of which 20,491 individuals were classified as of European American ancestry, 3998 of African American ancestry, and 1192 as other ancestry (17 unknown). A total of 5628 individuals were classified as dementia cases during the follow-up. The mean follow-up time and number of events for each age category are shown in Additional file 1: Table S1. In both samples, individuals with dementia were more likely to be female, have lower education, be non-smokers, and be older at baseline, at last follow-up, and at death (Table 1). The HRS participants were on average younger than the STR participants at first study participation, at end of follow-up, and at death, and had higher BMI. The mean BMI in each age category is shown in Additional file 1: Table S1.

**Table 1:** Descriptive statistics of the study population from the Swedish Twin Registry and the Health and Retirement Study

	The Swedish Twin Registry			The Health and Retirement Study		
	All	No dementia	Dementia	All	No dementia	Dementia
*N*	22,156	18,424	3732	25,698	20,070	5628
Female sex, *N* (%)	12,351 (55.75)	10,033 (54.46)	2318 (62.11)	14,273 (55.54)	10,875 (54.19)	3398 (60.38)
Low education, *N* (%)	9538 (43.05)	7319 (39.73)	2219 (59.46)	15,713 (61.14)	11,192 (55.76)	4521 (80.33)
Smokers, *N* (%)	12,076 (54.50)	10,381 (56.34)	1695 (45.42)	15,062 (58.61)	12,020 (59.89)	3042 (54.05)
Age at baseline, mean (SD)	66.75 (8.59)	66.40 (8.07)	68.44 (10.67)	62.92 (10.83)	60.69 (9.81)	70.90 (10.54)
Age at last follow-up, mean (SD)	79.19 (8.96)	77.83 (8.87)	85.91 (5.84)	75.07 (9.30)	73.82 (8.94)	79.55 (9.16)
Age at death, mean (SD)	83.88 (7.58)	82.76 (8.01)	86.44 (5.74)	81.43 (9.27)	79.15 (9.08)	85.23 (8.28)
BMI at baseline, mean (SD)	25.48 (3.77)	25.57 (3.83)	25.03 (3.48)	27.18 (5.18)	27.34 (5.22)	26.60 (5.01)

Descriptive statistics for all individuals, individuals without dementia, and individuals with dementia in the Swedish Twin Registry and the Health and Retirement Study. Statistics are presented as number (%) of individuals for categorical variables and mean level (SD) for continuous variables

*N* number, *SD* standard deviation, *BMI* body mass index

One SD higher PGS_BMI_ was significantly associated with higher BMI in both samples (*β* = 1.05, 95% confidence interval (CI) 0.96–1.14 in the STR; *β* = 1.22, 95% CI 1.13–1.32 in the HRS). Stratifying on ancestry in the HRS revealed stronger effects among individuals of European American descent (*β* = 1.30, 95% CI 1.20–1.49), but a substantial effect was present also in the African American descent sample (*β* = 0.82, 95% CI 0.56–1.08). Similarly, one SD higher PGS_AD_ was associated with higher odds of dementia in both samples (odds ratio (OR) = 1.49, 95% CI 1.39–1.59 in the STR; OR = 1.08, 95% CI 1.02–1.15 in the HRS). Stratifying on ancestry rendered similar results in the European American sample (OR = 1.10, 95% CI 1.03–1.18), but non-significant results among those of African American descent (OR = 1.03, 95% CI 0.91–1.17) in the HRS.

### Age-dependent effects of BMI

In the STR sample, a significant association between adulthood and midlife BMI and dementia was identified, with 5 units higher BMI associated with 15% (95% CI 7–24) higher risk of dementia when BMI was measured at age 35–49, and 11% (95% CI 1–22) higher risk when measured at age 50–64 (Table 2). The association then shifted to the inverse direction, with 5 units higher BMI associated, though not significantly, with 4% (95% CI − 1–10) lower risk of dementia when BMI was measured at age 65–79 and 10% (95% CI 0–19) when measured after age 80.

**Table 2:** Risk of incident dementia in relation to 5 units higher body mass index measured at different age categories in the Swedish Twin Registry

Age category	20–34	35–49	50–64	65–79	80+
*N*	8098	10,424	11,941	13,224	2565
Main model^a^	1.10 (0.98–1.23)	**1.15 (1.07–1.24)**	**1.11 (1.01–1.22)**	0.96 (0.90–1.01)	0.90 (0.81–1.00)
Main model, genotyped^a^	1.18 (0.96–1.44)	1.12 (0.98–1.26)	0.99 (0.87–1.12)	0.94 (0.85–1.03)	0.87 (0.75–1.01)
Adjusted for					
PGS_BMI_^b^	1.20 (0.98–1.47)	1.12 (0.99–1.28)	0.98 (0.86–1.12)	0.93 (0.84–1.02)	0.88 (0.75–1.03)
PGS_BMI_ and PGS_AD_^c^	1.14 (0.94–1.40)	1.13 (0.99–1.29)	0.98 (0.86–1.13)	0.94 (0.85–1.04)	0.88 (0.75–1.03)
By tertiles of PGS_BMI_^d^					
Lowest tertile	1.28 (0.91–1.80)	**1.38 (1.08–1.78)**	1.11 (0.87–1.42)	0.91 (0.76–1.09)	1.13 (0.86–1.47)
Middle tertile	1.20 (0.85–1.70)	1.12 (0.89–1.40)	0.91 (0.72–1.16)	0.87 (0.73–1.03)	0.79 (0.60–1.04)
Highest tertile	1.11 (0.79–1.56)	0.98 (0.79–1.20)	0.96 (0.78–1.19)	0.97 (0.84–1.13)	**0.70 (0.52–0.94)**
*p* value interaction	0.55	**0.04**	0.44	0.50	**0.01**
Co-twin control model^a^					
All twins	1.06 (0.75–1.48)	1.21 (0.97–1.50)	1.18 (0.94–1.49)	0.90 (0.77–1.05)	1.16 (0.82–1.64)
Dizygotic twins	1.06 (0.73–1.54)	1.25 (0.98–1.60)	1.24 (0.96–1.61)	0.93 (0.79–1.10)	1.15 (0.78–1.69)
Monozygotic twins	1.03 (0.47–2.27)	1.07 (0.68–1.68)	0.98 (0.59–1.63)	0.76 (0.50–1.15)	1.19 (0.53–2.65)

Hazard rate ratios (95% confidence intervals) of dementia in relation to 5 units higher body mass index measured at different age categories in the Swedish Twin Registry sample. All models are adjusted for age, sex, smoking, and education

*PGS* polygenic score, *BMI* body mass index, *AD* Alzheimer’s disease

^a^Dementia = BMI + age + sex + smoking + education

^b^Dementia = BMI + age + sex + smoking + education + PGS_BMI_

^c^Dementia = BMI + age + sex + smoking + education + PGS_BMI_ + PGS_AD_

^d^Dementia = BMI + age + sex + smoking + education + PGS_BMI_ + BMI*PGS_BMI_

In the HRS sample, no association was present between BMI measured at age 50–64 and dementia (Table 3). BMI measured at age 65–79 and 80 and above was associated with 9 (95% CI 4–14) and 11% (95% CI 5–17) lower risk of dementia, respectively.

**Table 3:** Risk of incident dementia in relation to 5 units higher body mass index measured at different age categories in the Health and Retirement Study

Age category	50–64	65–79	80+
	*N* = 15,375	*N* = 15,297	*N* = 5467
Main model^a^	0.97 (0.91–1.04)	**0.91 (0.86–0.96)**	**0.89 (0.83–0.95)**
Main model, genotyped^a^	0.95 (0.86–1.06)	0.91 (0.83–1.01)	0.85 (0.73–1.00)
Adjusted for			
PGS_BMI_^b^	0.96 (0.86–1.06)	0.91 (0.82–1.00)	**0.83 (0.72–0.97)**
PGS_BMI_ and PGS_AD_^c^	0.96 (0.86–1.06)	0.91 (0.83–1.00)	**0.83 (0.71–0.97)**
By tertiles of PGS for BMI^d^			
Lowest tertile	0.94 (0.78–1.13)	0.97 (0.83–1.13)	0.84 (0.64–1.10)
Middle tertile	0.92 (0.77–1.10)	0.87 (0.73–1.03)	**0.71 (0.52–0.95)**
Highest tertile	0.99 (0.84–1.16)	0.89 (0.76–1.05)	0.96 (0.77–1.19)
*p* value interaction	0.70	0.49	0.42

Hazard rate ratios (95% confidence intervals) of dementia in relation to 5 units higher body mass index measured at different age categories in the Health and Retirement Study sample. All models are adjusted for age, sex, smoking, and education

*PGS* polygenic score, *BMI* body mass index, *AD* Alzheimer’s disease

^a^Dementia = BMI + age + sex + smoking + education

^b^Dementia = BMI + age + sex + smoking + education + PGS_BMI_

^c^Dementia = BMI + age + sex + smoking + education + PGS_BMI_ + PGS_AD_

^d^Dementia = BMI + age + sex + smoking + education + PGS_BMI_ + BMI*PGS_BMI_

### Genetic influences on the association between BMI and dementia

In both samples, adjusting the models for the PGS_BMI_ and the PGS_AD_ had little effect on the associations of BMI measured at any age and dementia (Tables 2 and 3). Stratifying into tertiles of genetic susceptibility to higher BMI demonstrated an interaction between the PGS_BMI_ and BMI at age 35–49 in the STR sample, with 38% (95% CI 8–78) higher risk of dementia among those in the lowest tertile of the PGS_BMI_, and no association in the middle or highest PGS_BMI_ tertile. Co-twin control analyses in the STR sample showed a reduction in effect among monozygotic twins compared to dizygotic twins in the 35–49 age band, further indicating genetic influences. There was also an interaction between the PGS_BMI_ and BMI measured after age 80 in the STR sample, with 30% (95% CI 6–48) lower risk of dementia among those in the highest tertile of the PGS_BMI_. In the HRS, the interaction was not significant, and the estimate pattern was U-shaped with the strongest protective effect in the middle PGS_BMI_ tertile.

### Sensitivity analyses

When analyzing men and women separately, the effect estimates of 5 units higher BMI in adulthood and midlife on dementia risk were similar across gender (Additional file 1: Table S4). However, the inverse association between BMI at age 65–79 and 80 and above and dementia risk was stronger among women than men, with 5 units higher BMI being associated with 7–17% lower risk of dementia only among women. Stratifying on genetic predisposition to higher BMI also showed stronger associations among women than men, both for the increased risk of dementia associated with higher adulthood and early midlife BMI and the lower risk associated with higher late-life BMI.

Stratifying the HRS sample by ethnicity showed comparable results, though the associations between late-life BMI and dementia were generally stronger among those with European ancestry (Additional file 1: Table S5).

**Fig. 1:**
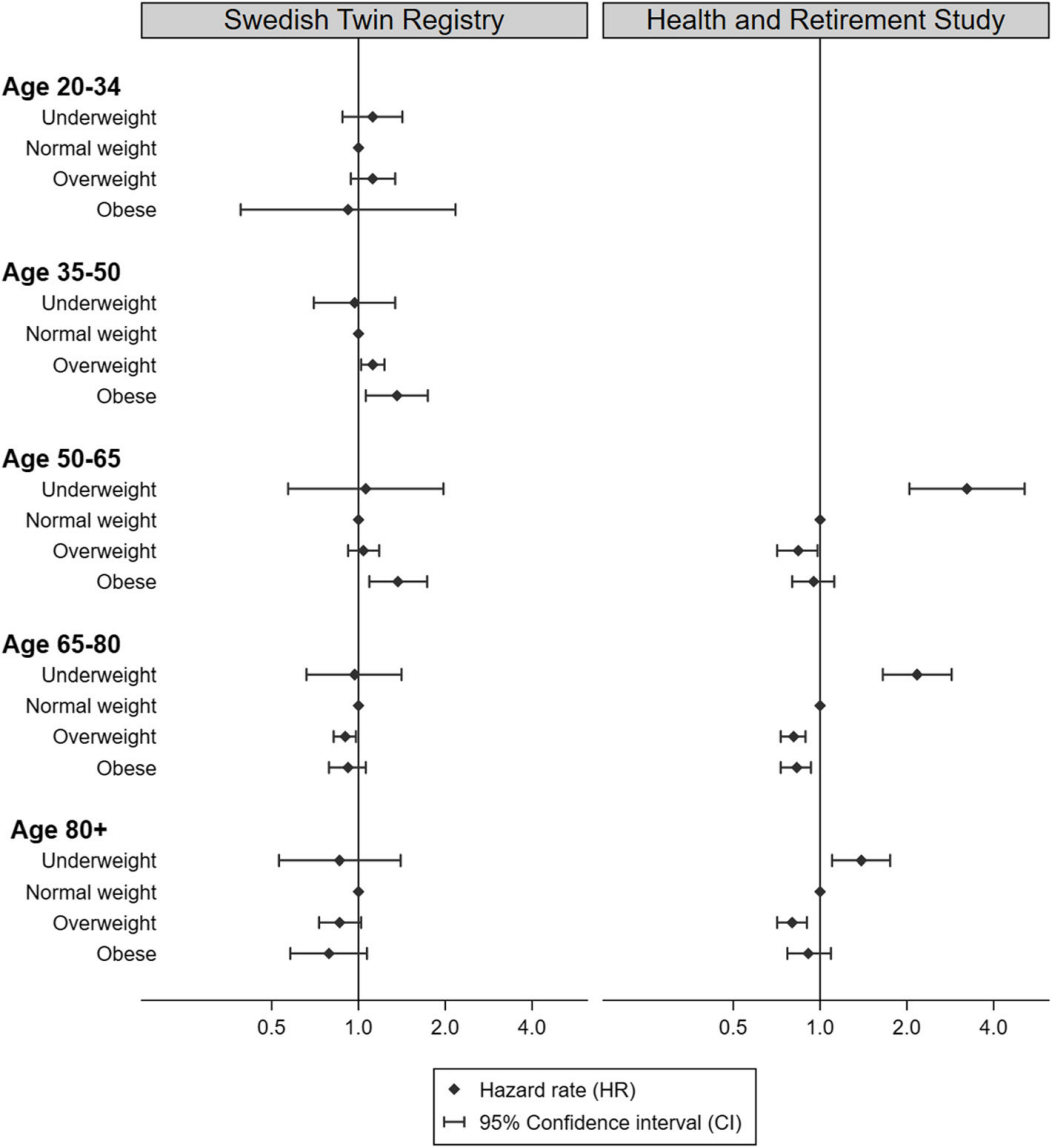
Hazard rate ratios and 95% confidence intervals of incident dementia in relation to being underweight, overweight, or obese at different age categories in the Swedish Twin Registry and the Health and Retirement Study. The models are adjusted for age, sex, smoking, and education

Categorizing BMI into weight categories revealed no increased risk of dementia among those who were underweight compared to normal weight at any age in the STR sample (Fig. 1 and Additional file 1: Table S6). However, in the HRS sample, being underweight was strongly associated with increased risk of dementia, with a hazard rate ratio (HR) of 3.23 (95% CI 2.04–5.12) at age 50–64, which decreased to 2.17 (95% CI 1.65–2.86) at age 65–79 and 1.39 (95% CI 1.10–1.75) after age 80 (Fig. 1 and Additional file 1: Table S6). In the STR sample, being overweight in adulthood and early midlife was associated with 12% (95% CI 2–23) higher risk of dementia, while being obese in adulthood or midlife was associated with 36 and 37% (95% CI 6–74 for adulthood and early midlife, 9–73 for midlife) higher risk, respectively. At age 65–79 and 80 and above, being overweight or obese was associated with 10–21% lower risk of dementia. In the HRS, the protective effect of being overweight was present already at age 50–64 and remained at 16–20% for overweight across all age groups. Stratifying on genetic predisposition to higher BMI generally showed a stronger negative effect of being underweight in early late-life and late-life among those with genetic predisposition to higher BMI in both samples (Additional file 1: Table S6).

Results from competing risk regression revealed cause-specific hazard rate ratios comparable to the HRs from the main model in the STR (Additional file 1: Table S7). While results were also comparable in the HRS, the general pattern when stratifying on PGS_BMI_ changed somewhat in the higher age categories, but the interaction remained non-significant.

## Discussion

Using longitudinal data from two large cohorts, the STR and HRS, we could confirm the age-specific effects of BMI on the risk of dementia, with higher BMI in adulthood and midlife being associated with higher risk of dementia in the STR, while a higher BMI in late-life was associated with a lower risk in both samples. In the STR, stratifying on genetic predisposition to higher BMI showed that the associations were only present among those with a BMI in opposite direction of their genetically predisposed BMI. The negative effect of a high BMI in adulthood and early midlife was present only among those with genetic predisposition to lower BMI, while the inverse association between late-life BMI and dementia risk was present only among those with genetic predisposition to a high BMI. Importantly, this indicates that the associations might be driven by environmental or other factors, rather than by BMI in itself. No clear difference based on genetic predisposition could be detected in the HRS.

To the best of our knowledge, very little work has been done on how genetic factors influence the association between BMI and dementia. One twin study demonstrated that the association between midlife overweight and dementia was substantially reduced within twin pairs, pointing to genetic confounding [25]. Similarly, we saw a substantial reduction in effect of adulthood and early midlife BMI within monozygotic compared to dizygotic twin pairs. Moreover, stratifying on genetic susceptibility to higher BMI indicated genetic influences, both an association between higher adulthood and early midlife BMI and dementia only among those with genetic predisposition to lower BMI, and a stronger negative effect of lower weight in late-life among those with genetic predisposition to higher BMI. Another way of utilizing genetic designs to understand disease biology and associations is Mendelian randomization studies, where genetic variants are used as instrumental variables to identify causal associations. Such studies have not found evidence of a causal effect of BMI on AD [26, 27], further strengthening our results indicating that both the negative effect of adulthood and early midlife BMI and the inverse association between late-life BMI and dementia are driven by environmental or other factors rather than by BMI in itself.

While the negative effect of a high midlife BMI on subsequent dementia risk is rather well-established [3], it is not without controversy. One study, based on almost 2 million individuals with a median age of 55 at baseline, showed that compared to individuals of normal weight, those who were overweight or obese in midlife had lower risk of developing dementia, while individuals who were underweight had increased risk [28]. The findings were replicated by Kivimäki et al., who found that individuals who were underweight in midlife had higher risk of dementia-related deaths, while those with overweight were of lower risk [29]. In line with these findings, we demonstrated a strong negative effect of being underweight in the HRS sample, the effect being stronger in the 50–64 age category, with a gradual decrease in the older age categories. In addition, a protective effect of being overweight was present already in the 50–64 age group in HRS and comparable in the older age groups. No such effect was seen in the STR sample, where obesity was associated with higher risk of dementia in both the 35–49 and 50–64 age groups. These and other difference in results from the HRS and the STR may be due to differences in the distribution of BMI, as both mean BMI and variability in BMI are higher in the HRS sample across all age categories, but especially so in the younger age groups. This difference in BMI distribution may be a result of cultural differences, such as diet and lifestyle. Further, as our findings indicate that the associations differ depending on genetic factors, differences in genetic background may also play a role in the observed differences.

Most evidence points to the obesity paradox being an effect of reverse causation, due to weight loss in the pre-clinical stage of dementia. A recent meta-analysis combined the results from 16 prospective studies of late-life BMI and the risk of incident dementia and found that the association between a higher BMI and lower dementia risk was only evident when follow-up was maximum 9 years [2]. Similarly, a recent review concluded that body weight starts to decline around 10 years prior to dementia diagnosis, and with a steeper decline in BMI trajectory among those who later develop dementia compared to those who do not [30]. A study by Russ et al. used data from over 30,000 individuals and found that those whose BMI started to decline earlier were at higher risk of dementia-related deaths compared to those whose BMI started to decline later [31]. The current study showed that at least in the Swedish sample, the inverse association between late-life BMI and dementia is most evident among those with genetic predisposition to a higher BMI. In addition, the increased risk of dementia among the underweight group in the HRS was the strongest among those with genetic predisposition to higher BMI. This may indicate that late-life weight loss is a stronger warning signal of underlying dementia among those genetically prone to a higher BMI.

We demonstrated sex differences in the findings, with the effect of late-life BMI as well as the interaction between adulthood and early midlife BMI and the PRS_BMI_ being present mainly among women. The basis for this sex difference remains to be investigated. One possibility is differences in body fat distribution between men and women, as where and what type of fat is stored affects the association between body fat and negative health outcomes [32]. It is also plausible that BMI is a better measure of overweight in women than in men in this sample, reflecting that men in this birth cohort may have had more physically demanding jobs. Thus, it may be that the higher BMI modeled as the predictor in this study in fact mirrors different aspects of body fat composition and overweight among men and women. Another potential explanation, especially for the differences in association between late-life BMI and dementia risk, is the male-female health-survival paradox, stating that women live longer despite higher rates of disability and poor health [33]. Thus, the effect may indicate an effect of poor health among women, leading to weight loss and signaling higher risk of dementia.

Strengths of the study are the well-powered longitudinal data collected over many years and the possibility to study the familial influences beyond those captured by the PGS through twin designs in the STR. However, we also acknowledge several limitations of the study and the data used. Firstly, BMI measurements from early adulthood through early midlife could not be studied in the HRS, hindering us from replicating those findings from the STR. In both samples, a combination of measured and self-reported height and weight was used. While self-reported weight, and especially retrospective, is not an ideal measure, it has been shown to be close to measured BMI and to represent an accurate mean level in the STR [34, 35]. Dementia diagnoses are not available in the HRS (other than for a small subsample), and we therefore used the Langa-Weir classifications [16]. It should be noted that the classification is based solely on the cognitive test results for self-responders and does not consider functional impairment. While the definition has been demonstrated to have a high accuracy [18], it is important to note that it does not represent clinical dementia diagnoses. Thus, some misclassification will remain and, assuming it is non-differential in relation to BMI, drive the estimates toward the null. For the STR data, clinical dementia diagnoses were not available for all twins, but could be complemented with register information. The specificity of the NPR and CDR has been shown to be excellent (98%), although the sensitivity is only 63% when both registers are combined. Thus, a proportion of individuals with dementia will remain undetected by the registers, introducing some extent of non-differential misclassification (assuming it is unrelated to BMI) and thus underestimation of true effects.

Studies of older individuals always entail methodological considerations related to attrition rate and survival bias, where those of poor health are less likely to be represented in the sample [36]. Moreover, in the context of BMI and risk of dementia, there is a risk that those at the high and low end of BMI are lost to follow-up due to poor health, morbidities, or death. While this would be less of a problem in the STR where individuals could be followed through register linkage, it may remain in the HRS. This could partly explain the difference in findings in the oldest age category, where the absence of inverse association between BMI and dementia among those in the highest PGS_BMI_ tertile may be an effect of higher dropout rate among that PGS category due to worse health. While the competing risk regression did not show evidence of survival bias in the STR, the findings were less stable in the HRS. In addition, while mortality data was available through register linkage for the STR, thus offering virtually complete coverage [37], mortality information for HRS participants was only available through informants and may thus not be as accurate. The competing risk regression in the HRS sample should therefore be interpreted with caution, as some attrition effects may remain. For the STR, GWAS summary statistics for BMI could be recalculated excluding the Swedish twin samples, but no such efforts were made for the PGS provided for HRS. As overlap between the GWAS and study sample leads to substantial overestimation of the effect of the PGS on the trait [19], the effect of introducing the PGS to the main model of the HRS data should be interpreted with caution. However, stratifying the sample into tertiles of genetic risk based on the PGS should be less affected by such bias. The PGS for AD in the HRS was only weakly associated with risk of dementia. This is likely due to a combination of less precise phenotype and the fact that the HRS pipeline uses all SNPs available when generating the score, rather than testing different *p* value cutoffs [21]. While that works well for most traits, it might be problematic for AD where, at least in the STR, restricting the PGS to SNPs with very low *p* values explained far more of the variance in AD.

Taken together, while combining the two large data sources does entail some limitations, it also presents opportunities where the sources complement each other, thus reducing the overall study limitations. Moreover, comparing the results while considering the limitations of each data source enables a more balanced interpretation of the study findings.

## Conclusions

This study confirms the age-specific effects of BMI on dementia risk and extends the current knowledge by demonstrating the importance of genetic influences. The increased risk for developing dementia among those with higher BMI in adulthood and early midlife was present only among those with genetic predisposition to lower BMI. Further, higher BMI in late-life was associated with lower risk of dementia, but here especially among those with genetic predisposition to higher BMI. More studies are warranted, but taken together, the present findings indicate that deviations from one’s genetically predisposed BMI might be an indicator of a higher dementia risk. Importantly, the findings point toward factors other than BMI in itself driving the association with dementia risk. In light of this, future efforts aimed at identifying these factors may help us identify targets for dementia risk reduction.

## Supplementary information

**Additional file 1.** Supplementary information about BMI data cleaning, Dementia data in the Swedish Twin Registry, and PGS calculation in the Swedish Twin Registry. **Table S1.** Age group characteristics for the analysis sample. **Table S2.** ICD codes used to identify dementia. **Table S3.** ATC-codes for identification of dementia medication. **Table S4.** Risk of incident dementia in relation to 5 units higher body mass index measured at different age categories in the Swedish Twin Registry and the Health and Retirement Study, stratified by sex. **Table S5.** Risk of incident dementia in relation to 5 units higher body mass index measured at different age categories in the Health and Retirement Study, stratified by ethnicity. **Table S6.** Risk of incident dementia in relation to being underweight, overweight, or obese at different age categories in the Swedish Twin Registry and the Health and Retirement Study. **Table S7.** Cause specific hazard rate ratios of dementia in relation to 5 units higher body mass index measured at different age categories in the Swedish Twin Registry and the Health and Retirement Study. **Figure S1.** Flow chart of the sample. **Figure S2.** Collections of BMI information in the Swedish Twin Registry.

## Data Availability

All codes used to generate analysis data and for conducting analyses are available upon request to the corresponding author. The STR data can be applied for at https://ki.se/en/research/swedish-twin-registry-for-researchers. The HRS and RAND files are public use datasets, available through registration at https://hrs.isr.umich.edu/data-products/access-to-public-data. The RAND HRS Longitudinal File 2014 (V3) was produced by the RAND Center for the Study of Aging, with funding from the National Institute on Aging and the Social Security Administration, Santa Monica, CA. Additional datasets from the Health and Retirement Study (Langa-Weir Classification of Cognitive Function (v.1.0) and Cross-Wave: Polygenic Score Data (PGS) (v.3.0)) are public use dataset, produced and distributed by the University of Michigan with funding from the National Institute on Aging (grant number NIA U01AG009740), Ann Arbor, MI.

## Abbreviations

AD: Alzheimer’s disease
ATC: Anatomical Therapeutic Chemical
BMI: Body mass index
CDR: Cause of Death Register
CI: Confidence interval
CIND: Cognitive impairment, no dementia
GWAS: Genome-wide association study
HR: Hazard rate ratio
HRS: Health and Retirement Study
ICD: International Classification of Diseases
NPR: National Patient Register
OR: Odds ratio
PDR: Prescribed Drug Register
PGS: Polygenic score
PGS_AD_: Polygenic score for Alzheimer’s disease
PGS_BMI_: Polygenic score for body mass index
SD: Standard deviation
SNP: Single nucleotide polymorphism
STR: Swedish Twin Registry
