# Supplementary material for: Age-dependent effects of body mass index across the adult life span on the risk of dementia: a cohort study with a genetic approach
Source: BMC Med. 2020 Jun 9;18:131. doi: 10.1186/s12916-020-01600-2 (PMC7282125; doi:10.1186/s12916-020-01600-2)
Supplement: Supplementary file 1 — Additional file 1. Supplementary information about BMI data cleaning, Dementia data in the Swedish Twin Registry, and PGS calculation in the Swedish Twin Registry. Table S1. Age group characteristics for the analysis sample. Table S2. ICD codes used to identify dementia. Table S3. ATC-codes for identification of dementia medication. Table S4. Risk of incident dementia in relation to 5 units higher body mass index measured at different age categories in the Swedish Twin Registry and the Health and Retirement Study, stratified by sex. Table S5. Risk of incident dementia in relation to 5 units higher body mass index measured at different age categories in the Health and Retirement Study, stratified by ethnicity. Table S6. Risk of incident dementia in relation to being underweight, overweight, or obese at different age categories in the Swedish Twin Registry and the Health and Retirement Study. Table S7. Cause specific hazard rate ratios of dementia in relation to 5 units higher body mass index measured at different age categories in the Swedish Twin Registry and the Health and Retirement Study. Figure S1. Flow chart of the sample. Figure S2. Collections of BMI information in the Swedish Twin Registry. [file 12916_2020_1600_MOESM1_ESM.docx]

**Supplementary section**

**BMI data cleaning**

Swedish Twin Registry

The height and weight data in the Swedish Twin Registry (STR) have either been measured by trained research nurses, self-reported, or self-reported retrospectively, in centimeters and kilos as relevant. Although there are problems with self-reports, especially retrospectively, we have shown that these measures are fair in the current setting(1, 2). Height and weight have been thoroughly examined for outliers by taking advantage of the repeated measures of the variables of interest. The height, weight, and BMI data have been analyzed for outliers by identifying outliers quantitatively but also by visually analyzing the trajectories by plotting all individuals’ trajectories. For persons with missing data or with unrealistic values, height was imputed if there were two or more measures of height available from other waves. If the available height measures were exactly the same, that number was imputed. If the available measures differed, the mean of the other measures were used. As it is not uncommon that height declines in old age, that was taken into account when height was imputed in old age. In other words, data were imputed to follow the overall trend in height. Missing values and unrealistic values of weight were not imputed as weight might fluctuate.

BMI was calculated as kg/m2. Unrealistic drops or increases during a short time period (e.g. 10 units increase in BMI over two years and then two years later being back at the BMI before the increase), were set to missing, but otherwise BMI was allowed to vary. BMI measures below 15 and above 55 were dropped.

Health and Retirement Study

Height and weight for each study wave were retrieved and converted to kilograms and meters. Height was measured at several occasions. To remove outliers, mean and standard deviation (SD) in height was calculated for each individual based on their unique height measures. For all with SD >0.05 (n=1947), the following steps were taken: individuals with less than 3 height measurements were removed, and height measurements more than 1 SD from the individual’s mean were set to missing. Mean and SD for each individual were then calculated again based on the remaining measures, and again, for all with SD >0.05 all individuals with less than 3 height measurements remaining were removed, and height measurements more than 1 SD from the individual’s mean were set to missing. Mean and SD was then calculated again, based on remaining measures, after which individuals with SD >0.05 were removed. In addition, individuals with mean height below 1.47 or above 2.10 were removed. In total, this led to dropping 1,486 observations (n=1186 due to too much variation across measures; n=300 with height below 1.47 or above 2.10). For the remaining observations, mean height was used to calculate BMI across all waves.

BMI was calculated as kg/m2. As weight is expected to have high variability in this age group, minimal exclusions were made, only dropping BMI measures <15. No exclusions were made in the higher BMI range in the HRS sample to account for the higher mean BMI level and standard deviation seen in the sample.

**Dementia data in the Swedish Twin Registry**

**Clinical dementia ascertainment**

The SATSA, OTCTO-Twin, GENDER, and HARMONY studies included clinical dementia ascertainment(3). In SATSA, OCTO-Twin, and GENDER, the in-person testing phases included cognitive screening using the Mini-Mental State Examination (4) and additional cognitive tests. In HARMONY, the TELE screening (5) was administered during the telephone interview, complemented with informant interviews with the Blessed Dementia Rating Scale (BDRS)(6) for those who performed poorly on the TELE. In HARMONY and the first three in-person testing phases of SATSA, all individuals suspected of dementia, as well as their co-twins, were referred to a clinical examination including cognitive testing, physical and neurological work-ups, reviews of medical records, informant interviews, and laboratory tests. In OCTO-Twin, GENDER, and from the fifth in-person testing phase in SATSA, dementia diagnoses were based on the extensive cognitive testing, review of medical records, and the research nurse’s evaluation. For all sub-studies, final dementia diagnosis was set at multidisciplinary consensus conferences, according to DSM-III-R(7) or DSM-IV(8) criteria, with further differential diagnosis for Alzheimer’s disease, vascular dementia, Lewy Body dementia, and other causes of dementia. Those with psychiatric disorders or intellectual disability were excluded.

**Dementia from national registers**

Through the 10-digit personal identification number assigned to all residents in Sweden, the STR is linked to several population-based registries. Dementia diagnoses were retrieved from the National Patient Registry (NPR) and the Cause of Death Registry (CDR), and information about medication from the Prescribed Drug Registry (PDR).

The NPR, initiated in 1964 with nationwide coverage since 1987, includes information about 99% of all in-patient care at hospitals in Sweden(9). For each hospitalization, the primary diagnosis together with up to 20 additional diagnoses is recorded according to International Classification of Diseases (ICD) codes. Since 2001, the NPR also covers outpatient specialist care. Since 1961, the CDR includes information about underlying and contributory causes of death for all Swedish residents, also reported according to ICD codes(10). Data from NPR and CDR are currently available through the end of 2016. Both primary and additional diagnoses from the NPR, as well as underlying and contributing causes of death from the CDR were used as criteria for disease. ICD codes used to retrieve dementia diagnoses are reported in Table S2. Codes were chosen to encompass chronic, progressive impairment of cognitive function.

Since 2005, The PDR contains information on all dispensed prescriptions classified according to Anatomical Therapeutic Chemical (ATC) codes(11). The register is currently updated through the end of 2017, but for this study only information through 2016 was included to correspond to the information from the NPR and CDR. ATC codes for dementia medication are reported in Table S3.

**PGS calculation in the Swedish Twin Registry**

Twins in the STR were genotyped on Human OmniExpress (TwinGene) or Illumina PsychArray (SATSA, GENDER, HARMONY). The data were imputed against the 1000 Genomes Project phase 1 version 3 data(12). Genetic variants present on both genotyping arrays with minor allele frequency >0.01 and good imputation quality (based on info score >0.8) were selected for PGS computation. To obtain independent genetic variants from the GWAS summary statistics, LD-clumping based on 1000 Genomes Project phase 1 version 3 data(12) was performed setting R2 to 0.01. The scores were then computed in Plink 1.9 by, for each individual in the sample, summing the number of risk alleles at each independent single nucleotide polymorphism (SNP) across the genome, weighted by the SNP effect size from the GWAS of the trait. Nine sets of PGSs were computed for respective trait by including independent SNPs reaching a significance level ranging from p=5x10^-8^ to p=1 in the GWAS. The trait variance explained by each PGS was calculated by comparing the R^2^ (BMI) or Nagelkerke R^2^ (AD) from a full model including both the PGS and covariates (age, sex, and 3 principal components) as predictors to that of a reduced model including only the covariates. The PGS_BMI_ using all independent SNPs with p<0.5 best explained variance in BMI and was used in subsequent analyses. For AD, the PGS_AD_ using independent SNPs with p<10^-5^ had the best explanatory ability. Prior to analyses, the PGSs were adjusted for ancestry by residualizing on the first three principal components in the STR samples and the first 5 in the HRS samples (to account for higher heterogeneity within the sample). The PGSs were then standardized within genotyping array (STR) or race (HRS) to facilitate easier interpretation of results.

**Table S1: Age group characteristics for the analysis sample**

| **Age category** | **20-34** | **35-49** | **50-64** | **65-79** | **80+** |
| --- | --- | --- | --- | --- | --- |
| **The Swedish Twin Registry** |  |  |  |  |  |
| Number of individuals | 8098 | 10424 | 11941 | 13224 | 2565 |
| Number of BMI measures | 8098 | 15909 | 18751 | 17654 | 4391 |
| Mean BMI (SD) | 21.8 (2.4) | 23.6 (2.9) | 25.3 (3.6) | 25.7 (3.7) | 24.7 (3.8) |
| Mean age (SD) | 26.6 (3.1) | 42.5 (3.5) | 57.2 (4.1) | 70.8 (4.2) | 84.1 (3.4) |
| Mean follow-up time (range) | 51.7 (32-83) | 39.6 (11-68) | 18.9 (17-52) | 12.1 (1-43) | 7.5 (1-23) |
| Dementia events | 1526 | 2540 | 1223 | 2394 | 667 |
| Number of genotyped individuals | 4630 | 4240 | 8823 | 6239 | 1057 |
| **The Health and Retirement Study** |  |  |  |  |  |
| Number of individuals |  |  | 15 375 | 15 297 | 5 467 |
| Number of BMI measures |  |  | 64 670 | 50 759 | 13 306 |
| Mean BMI (SD) |  |  | 28.1 (5.5) | 27.3 (5.0) | 25.2 (4.3) |
| Mean age (SD) |  |  | 58.5 (3.8) | 71.8 (4.2) | 84.6 (3.7) |
| Mean follow-up time (range) |  |  | 12.6 (0-23) | 7.8 (0-23) | 4.8 (0-21) |
| Dementia events |  |  | 952 | 2072 | 1383 |
| Number of genotyped individuals |  |  | 8727 | 7549 | 2002 |

**Table S2: ICD codes used to identify dementia**

| **ICD-7** | **ICD-8** | **ICD-9*** | **ICD-10** |
| --- | --- | --- | --- |
| (used before 1969) | (used 1969-1986) | (used 1987-1996) | (used 1997 and onwards) |
| **304** Senile psychosis | **290** Senile and presenile dementia | **290** Senile and presenile organic psychotic condition | **F00** Dementia in Alzheimer's disease |
| **305** Presenile psychosis | **293.0** Cerebral arteriosclerosis | **294B/ 294.1** Dementia in conditions classified elsewhere | **F01** Vascular dementia |
| **306** Psychosis with cerebral arteriosclerosis | **293.1** Other cerebrovascular disturbances | **331A/ 331.0** Alzheimer's disease | **F02** Dementia in other diseases classified elsewhere |
|  |  | **331B/ 331.1** Pick's disease | **F03** Unspecified dementia |
|  |  | **331C/ 331.2** Senile degeneration of brain | **F051** Delirium superimposed on dementia |
|  |  | **331X/ 331.9** Cerebral degeneration, unspecified | **G30** Alzheimer's disease |
|  |  |  | **G311** Senile degeneration of brain, not elsewhere classified |
|  |  |  | **G318A** Other specified degenerative diseases of nervous system: Lewy body dementia |

* In the Swedish adaptation of ICD-9, the 4^th^ digit was replaced with a letter. While the NPR used the Swedish version, the international ICD version was used in CDR(10), and both versions are therefore reported here.

**Table S3: ATC-codes for identification of dementia medication**

| **N06DA** Anticholinesterases |
| --- |
| N06DA02 Donepezil |
| N06DA03 Rivastigmine |
| N06DA04 Galantamine |
| (N06DA01 Tacrine and N06DA05 Ipidacrine not prescribed in Sweden) |
| **N06DX** Other anti-dementia drugs |
| N06DX01 Memantine |
| (N06DX02 Ginkgo folium not prescribed in Sweden) |

**Table S4:** **Risk of incident dementia in relation to 5 units higher body mass index measured at different age categories in the Swedish Twin Registry and the Health and Retirement Study, stratified by sex**

1. **Men**

| **Study sample** | **The Swedish Twin Registry** | | | | | **The Health and Retirement Study** | | |
| --- | --- | --- | --- | --- | --- | --- | --- | --- |
| **Age Category** | **20-34** | **35-49** | **50-64** | **65-79** | **80+** | **50-64** | **65-79** | **80+** |
| **N** | 3246 | 4369 | 5098 | 6087 | 1024 | 7084 | 6741 | 2053 |
| **Main model** | 1.15 (0.92-1.43) | 1.17 (1.02-1.35) | 1.04 (0.86-1.27) | 1.01 (0.90-1.12) | 1.10 (0.91-1.33) | 0.97 (0.86-1.10) | 0.98 (0.89-1.08) | 0.93 (0.81-1.07) |
| **Main model, genotyped** | 1.38 (0.94-2.02) | 1.16 (0.92-1.47) | 1.00 (0.77-1.29) | 1.05 (0.89-1.24) | 1.01 (0.79-1.31) | 0.92 (0.76-1.11) | 1.07 (0.90-1.27) | 1.06 (0.77-1.46) |
| **By tertiles of PGS for BMI** |  |  |  |  |  |  |  |  |
| Lowest tertile | 1.64 (0.84-3.20) | 1.23 (0.82-1.86) | 0.99 (0.65-1.51) | 0.96 (0.70-1.32) | 1.32 (0.87-1.99) | 0.84 (0.58-1.22) | 1.20 (0.87-1.66) | 0.91 (0.51-1.64) |
| Middle tertile | 1.39 (0.80-2.43) | 1.01 (0.68-1.50) | 0.96 (0.59-1.58) | 0.95 (0.72-1.25) | 0.94 (0.56-1.58) | 0.88 (0.63-1.22) | 1.07 (0.77-1.49) | 0.93 (0.48-1.80) |
| Highest tertile | 1.16 (0.57-2.34) | 1.22 (0.78-1.92) | 1.04 (0.66-1.63) | 1.18 (0.90-1.54) | 0.79 (0.48-1.30) | 0.98 (0.72-1.34) | 0.95 (0.72-1.25) | 1.23 (0.84-1.80) |
| P-value interaction | 0.48 | 0.97 | 0.87 | 0.30 | 0.11 | 0.52 | 0.27 | 0.37 |

1. **Women**

| **Study sample** | **The Swedish Twin Registry** | | | | | **The Health and Retirement Study** | | |
| --- | --- | --- | --- | --- | --- | --- | --- | --- |
| **Age Category** | **20-34** | **35-49** | **50-64** | **65-79** | **80+** | **50-64** | **65-79** | **80+** |
| **N** | 4852 | 6055 | 6843 | 7137 | 1541 | 8291 | 8556 | 3414 |
| **Main model** | 1.08 (0.94-1.23) | 1.15 (1.05-1.25) | 1.14 (1.02-1.26) | 0.93 (0.87-1.00) | 0.83 (0.73-0.94) | 0.97 (0.90-1.06) | 0.88 (0.82-0.93) | 0.88 (0.81-0.95) |
| **Main model, genotyped** | 1.11 (0.88-1.40) | 1.10 (0.95-1.27) | 0.96 (0.83-1.12) | 0.88 (0.78-0.99) | 0.80 (0.66-0.97) | 0.98 (0.87-1.10) | 0.85 (0.76-0.96) | 0.79 (0.66-0.95) |
| **By tertiles of PGS for BMI** |  |  |  |  |  |  |  |  |
| Lowest tertile | 1.17 (0.79-1.74) | 1.46 (1.07-1.98) | 1.11 (0.82-1.52) | 0.87 (0.70-1.09) | 1.04 (0.74-1.46) | 0.99 (0.80-1.21) | 0.89 (0.74-1.07) | 0.81 (0.59-1.11) |
| Middle tertile | 1.16 (0.76-1.77) | 1.16 (0.89-1.53) | 0.88 (0.68-1.14) | 0.83 (0.67-1.02) | 0.72 (0.51-1.02) | 0.96 (0.78-1.18) | 0.79 (0.65-0.96) | 0.64 (0.47-0.88) |
| Highest tertile | 1.08 (0.73-1.60) | 0.91 (0.72-1.15) | 0.92 (0.73-1.17) | 0.89 (0.74-1.08) | 0.67 (0.46-0.96) | 0.98 (0.81-1.20) | 0.86 (0.70-1.05) | 0.88 (0.68-1.14) |
| P-value interaction | 0.79 | 0.02 | 0.39 | 0.82 | 0.07 | 0.99 | 0.80 | 0.68 |

Hazard rate ratios (95% confidence intervals) of dementia in relation to 5 units higher body mass index measured at different age categories in men and women from the Swedish Twin Registry. All models are adjusted for age, smoking, and education.

**Table S5: Risk of incident dementia in relation to 5 units higher body mass index measured at different age categories in the Health and Retirement Study, stratified by ethnicity**

| **Ethnicity** | **European ancestry** | | | **African ancestry** | | |
| --- | --- | --- | --- | --- | --- | --- |
| **Age Category** | **50-64** | **65-79** | **80+** | **50-64** | **65-79** | **80+** |
| **N** | 11917 | 13044 | 4839 | 2615 | 1820 | 521 |
| **Main model** | 1.01 (0.92-1.11) | 0.90 (0.84-0.96) | 0.85 (0.79-0.93) | 0.92 (0.82-1.02) | 0.89 (0.81-0.98) | 1.00 (0.88-1.13) |
| **Main model, genotyped** | 0.92 (0.79-1.07) | 0.91 (0.81-1.03) | 0.85 (0.71-1.01) | 0.99 (0.86-1.13) | 0.91 (0.77-1.06) | 0.90 (0.65-1.24) |
| **Adjusted for PGS for:** |  |  |  |  |  |  |
| BMI | 0.91 (0.77-1.06) | 0.90 (0.79-1.02) | 0.82 (0.69-0.98) | 0.99 (0.87-1.14) | 0.91 (0.78-1.07) | 0.90 (0.65-1.24) |
| BMI and AD | 0.91 (0.77-1.06) | 0.90 (0.79-1.02) | 0.82 (0.69-0.98) | 0.99 (0.87-1.14) | 0.91 (0.78-1.07) | 0.90 (0.65-1.24) |
| **By tertiles of PGS for BMI** |  |  |  |  |  |  |
| Lowest tertile | 0.96 (0.70-1.30) | 0.93 (0.75-1.16) | 0.81 (0.59-1.11) | 0.89 (0.70-1.14) | 0.96 (0.75-1.22) | 0.94 (0.58-1.54) |
| Middle tertile | 0.78 (0.61-1.00) | 0.81 (0.65-1.01) | 0.69 (0.49-0.96) | 1.09 (0.88-1.34) | 0.99 (0.76-1.29) | 0.80 (0.43-1.47) |
| Highest tertile | 0.97 (0.77-1.22) | 0.95 (0.78-1.15) | 0.98 (0.77-1.24) | 1.03 (0.80-1.31) | 0.78 (0.55-1.09) | 0.94 (0.54-1.64) |
| P-value interaction | 0.84 | 0.86 | 0.33 | 0.41 | 0.33 | 0.99 |

Hazard rate ratios (95% confidence intervals) of dementia in relation to 5 units higher body mass index measured at different age categories in the Health and Retirement Study sample, stratified by ethnicity. All models are adjusted for age, sex, smoking, and education.

**Table S6:** **Risk of incident dementia in relation to being underweight, overweight, or obese at different age categories in the Swedish Twin Registry and the Health and Retirement Study**

| **Study sample** | **The Swedish Twin Registry** | | | | | **The Health and Retirement Study** | | |
| --- | --- | --- | --- | --- | --- | --- | --- | --- |
| **Age Category** | **20-34** | **35-49** | **50-64** | **65-79** | **80+** | **50-64** | **65-79** | **80+** |
| **N underweight** | 504 | 207 | 129 | 199 | 107 | 262 | 377 | 293 |
| **N normal weight** | 6867 | 7694 | 6770 | 6326 | 1488 | 5985 | 6340 | 3079 |
| **N overweight** | 684 | 2804 | 5285 | 6008 | 1028 | 8730 | 8006 | 2344 |
| **N obese** | 43 | 378 | 1315 | 1604 | 261 | 5902 | 4562 | 881 |
| **Main model** |  |  |  |  |  |  |  |  |
| Underweight | 1.12 (0.88-1.42) | 0.97 (0.70-1.34) | 1.06 (0.57-1.97) | 0.97 (0.66-1.41) | 0.86 (0.53-1.40) | 3.23 (2.04-5.12) | 2.17 (1.65-2.86) | 1.39 (1.10-1.75) |
| Overweight | 1.12 (0.94-1.34) | 1.12 (1.02-1.23) | 1.04 (0.92-1.18) | 0.90 (0.82-0.98) | 0.86 (0.73-1.02) | 0.84 (0.71-0.98) | 0.81 (0.73-0.89) | 0.80 (0.71-0.90) |
| Obese | 0.92 (0.39-2.17) | 1.36 (1.06-1.74) | 1.37 (1.09-1.73) | 0.92 (0.79-1.06) | 0.79 (0.58-1.07) | 0.95 (0.80-1.12) | 0.83 (0.73-0.93) | 0.91 (0.77-1.09) |
| **Main model, genotyped** |  |  |  |  |  |  |  |  |
| Underweight | 1.22 (0.84-1.79) | 0.86 (0.47-1.60) | 1.36 (0.69-2.66) | 1.08 (0.49-2.36) | 0.93 (0.44-1.96) | 1.65 (0.69-3.94) | 2.73 (1.59-4.69) | 1.86 (1.03-3.35) |
| Overweight | 1.11 (0.81-1.52) | 1.06 (0.89-1.25) | 0.85 (0.70-1.02) | 0.93 (0.80-1.08) | 0.80 (0.63-1.03) | 0.76 (0.60-0.96) | 0.73 (0.61-0.87) | 0.78 (0.61-0.99) |
| Obese | 1.82 (0.51-6.55) | 1.20 (0.78-1.85) | 1.12 (0.81-1.56) | 0.86 (0.68-1.08) | 0.61 (0.39-0.96) | 0.82 (0.64-1.05) | 0.82 (0.67-1.01) | 0.81 (0.56-1.18) |
| **By tertiles of PGS for BMI** |  |  |  |  |  |  |  |  |
| **Lowest tertile** |  |  |  |  |  |  |  |  |
| Underweight | 1.40 (0.76-2.58) | 0.71 (0.27-1.85) | 1.51 (0.61-3.75) | 1.14 (0.48-2.70) | 0.98 (0.41-2.31) | 1.42 (0.43-4.69) | 2.39 (1.14-5.03) | 1.64 (0.61-4.39) |
| Overweight | 0.85 (0.42-1.70) | 1.15 (0.84-1.58) | 1.01 (0.73-1.39) | 0.95 (0.74-1.22) | 0.98 (0.64-1.52) | 0.79 (0.54-1.16) | 0.82 (0.61-1.11) | 0.80 (0.52-1.25) |
| Obese | 3.01 (1.42-6.37) | 2.20 (0.63-7.65) | 1.61 (0.79-3.25) | 0.90 (0.56-1.44) | 1.53 (0.71-3.31) | 0.94 (0.61-1.46) | 0.90 (0.62-1.32) | 0.77 (0.39-1.52) |
| **Middle tertile** |  |  |  |  |  |  |  |  |
| Underweight | 1.10 (0.59-2.08) | 1.07 (0.41-2.80) | 0.93 (0.50-1.73) | - | - | 1.49 (0.20-10.9) | 3.24 (1.18-8.89) | 2.42 (1.10-5.35) |
| Overweight | 1.38 (0.85-2.24) | 1.04 (0.77-1.40) | 0.73 (0.52-1.02) | 0.84 (0.66-1.07) | 0.67 (0.43-1.03) | 0.77 (0.52-1.16) | 0.61 (0.46-0.82) | 0.71 (0.49-1.05) |
| Obese | - | 1.53 (0.81-2.91) | 1.13 (0.67-1.91) | 0.64 (0.42-0.98) | 0.54 (0.27-1.07) | 0.74 (0.47-1.15) | 0.85 (0.62-1.17) | 0.63 (0.33-1.23) |
| **Highest tertile** |  |  |  |  |  |  |  |  |
| Underweight | 1.17 (0.59-2.32) | 0.97 (0.21-4.34) | 1.88 (0.27-13.0) | 2.16 (0.46-10.2) | 3.16 (0.64-15.7) | 3.39 (1.38-8.32) | 3.39 (1.02-11.2) | 1.58 (0.33-7.48) |
| Overweight | 1.08 (0.66-1.77) | 1.00 (0.75-1.32) | 0.81 (0.59-1.12) | 0.98 (0.74-1.30) | 0.75 (0.47-1.20) | 0.70 (0.45-1.10) | 0.76 (0.55-1.05) | 0.77 (0.50-1.20) |
| Obese | 1.47 (0.17-12.6) | 0.90 (0.49-1.65) | 0.95 (0.58-1.58) | 0.96 (0.67-1.37) | 0.32 (0.13-0.78) | 0.79 (0.51-1.21) | 0.71 (0.49-1.02) | 1.02 (0.57-1.81) |

Hazard rate ratios (95% confidence intervals) of dementia among those who are underweight, overweight, or obese, compared to those of normal weight at different age categories in the Swedish Twin Registry and the Health and Retirement Study. All models are adjusted for age, sex, smoking, and education.

**Table S7: Cause specific hazard rate ratios of dementia in relation to 5 units higher body mass index measured at different age categories in the Swedish Twin Registry and the Health and Retirement Study**

| **Study sample** | **The Swedish Twin Registry** | | | | | **The Health and Retirement Study** | | |
| --- | --- | --- | --- | --- | --- | --- | --- | --- |
| **Age Category** | **20-34** | **35-49** | **50-64** | **65-79** | **80+** | **50-64** | **65-79** | **80+** |
| **N** | 8098 | 10424 | 11941 | 13224 | 2565 | 15 375 | 15 297 | 5 467 |
| **Main model** | 1.06 (0.95-1.19) | 1.07 (1.00-1.15) | 1.05 (0.96-1.15) | 0.92 (0.87-0.98) | 0.90 (0.81-1.00) | 0.94 (0.88-1.00) | 0.86 (0.83-0.90) | 0.87 (0.82-0.93) |
| **Main model, genotyped** | 1.12 (0.92-1.35) | 1.06 (0.95-1.19) | 0.95 (0.84-1.07) | 0.91 (0.83-1.00) | 0.84 (0.72-0.98) | 0.96 (0.87-1.06) | 0.87 (0.79-0.95) | 0.91 (0.77-1.08) |
| **Adjusted for PGS for:** |  |  |  |  |  |  |  |  |
| BMI | 1.16 (0.96-1.41) | 1.09 (0.96-1.23) | 0.95 (0.83-1.08) | 0.91 (0.83-1.00) | 0.87 (0.74-1.02) | 0.97 (0.87-1.07) | 0.87 (0.79-0.95) | 0.91 (0.77-1.08) |
| BMI and AD | 1.10 (0.91-1.34) | 1.07 (0.95-1.22) | 0.94 (0.82-1.07) | 0.91 (0.83-1.00) | 0.87 (0.74-1.02) | 0.97 (0.87-1.07) | 0.87 (0.79-0.95) | 0.91 (0.77-1.08) |
| **By tertiles of PGS for BMI** |  |  |  |  |  |  |  |  |
| Lowest tertile | 1.27 (0.92-1.76) | 1.27 (1.00-1.61) | 1.04 (0.82-1.31) | 0.87 (0.73-1.03) | 1.07 (0.81-1.39) | 0.90 (0.75-1.08) | 0.91 (0.78-1.06) | 0.90 (0.67-1.21) |
| Middle tertile | 1.20 (0.85-1.70) | 1.10 (0.88-1.38) | 0.90 (0.72-1.13) | 0.86 (0.73-1.02) | 0.77 (0.58-1.01) | 0.99 (0.84-1.17) | 0.86 (0.73-1.02) | 0.98 (0.73-1.32) |
| Highest tertile | 1.01 (0.74-1.38) | 0.95 (0.78-1.14) | 0.93 (0.76-1.13) | 0.96 (0.83-1.11) | 0.73 (0.54-0.98) | 0.98 (0.83-1.16) | 0.83 (0.71-0.96) | 0.86 (0.66-1.12) |
| P-value interaction | 0.30 | 0.05 | 0.52 | 0.36 | 0.05 | 0.50 | 0.36 | 0.83 |

Cause-specific hazard rate ratios (95% confidence intervals) of dementia in relation to 5 units higher body mass index measured at different age categories in the Swedish Twin Registry and the Health and Retirement Study. Dementia was treated as the cause-specific outcome and death as the competing event. All models are adjusted for age, sex, smoking, and education.

**Figure S1: Flow chart of the sample**

1. **The Swedish Twin Registry**

Swedish Twin Registry: participants in SATSA, OCTO-Twin, GENDER, HARMONY, or TwinGene

N= 24 823

(n= 12 636 with genotype data)

Missing other covariates (education or smoking)

N= 63

Diagnosed with early onset dementia

N= 21

Screening dementia suspect, but declined follow-up

N= 1509

Dementia at first BMI measure

N= 100

<60 at last follow-up

N= 385

No height and weight info

N= 577

No dementia information

N= 12

**Analysis sample**

**N= 22 156**

**(n=12 060 with genotype info)**

1. **The Health and Retirement Study**

The Health and Retirement Study

N= 37 494

(n= 15 190 with genotype data)

**Analysis sample**

**N= 25 698**

**(n=** **11 453 with genotype info)**

No dementia information

N= 1 974

No height and weight info

N= 1 785

<60 at last follow-up

N= 7 827

Missing other covariates (education or smoking)

N= 210

**Figure S2: Collections of BMI information in the Swedish Twin Registry**

**References**

1. Dahl AK, Reynolds CA. Accuracy of recalled body weight--a study with 20-years of follow-up. Obesity (Silver Spring). 2013;21(6):1293-8.

2. Dahl AK, Hassing LB, Fransson EI, Pedersen NL. Agreement between self-reported and measured height, weight and body mass index in old age--a longitudinal study with 20 years of follow-up. Age Ageing. 2010;39(4):445-51.

3. Gatz M, Fratiglioni L, Johansson B, Berg S, Mortimer JA, Reynolds CA, et al. Complete ascertainment of dementia in the Swedish Twin Registry: the HARMONY study. Neurobiol Aging. 2005;26(4):439-47.

4. Folstein MF, Folstein SE, McHugh PR. "Mini-mental state". A practical method for grading the cognitive state of patients for the clinician. J Psychiatr Res. 1975;12(3):189-98.

5. Gatz M, Reynolds CA, John R, Johansson B, Mortimer JA, Pedersen NL. Telephone screening to identify potential dementia cases in a population-based sample of older adults. Int Psychogeriatr. 2002;14(3):273-89.

6. Blessed G, Tomlinson BE, Roth M. The association between quantitative measures of dementia and of senile change in the cerebral grey matter of elderly subjects. Br J Psychiatry. 1968;114(512):797-811.

7. American Psychiatric Association. Diagnostic and statistical manual of mental disorders : DSM-III-R: American Psychiatric Association; 1987.

8. American Psychiatric Association. Diagnostic and statistical manual of mental disorders : DSM-IV: American Psychiatric Association; 1994.

9. Ludvigsson JF, Andersson E, Ekbom A, Feychting M, Kim JL, Reuterwall C, et al. External review and validation of the Swedish national inpatient register. BMC Public Health. 2011;11:450.

10. Brooke HL, Talback M, Hornblad J, Johansson LA, Ludvigsson JF, Druid H, et al. The Swedish cause of death register. European journal of epidemiology. 2017;32(9):765-73.

11. Wallerstedt SM, Wettermark B, Hoffmann M. The First Decade with the Swedish Prescribed Drug Register - A Systematic Review of the Output in the Scientific Literature. Basic Clin Pharmacol Toxicol. 2016;119(5):464-9.

12. 1000 Genomes Project Consortium, Abecasis GR, Auton A, Brooks LD, DePristo MA, Durbin RM, et al. An integrated map of genetic variation from 1,092 human genomes. Nature. 2012;491(7422):56-65.
